# Fine Particulate Air Pollution and Hospital Emergency Room Visits for Respiratory Disease in Urban Areas in Beijing, China, in 2013

**DOI:** 10.1371/journal.pone.0153099

**Published:** 2016-04-07

**Authors:** Qin Xu, Xia Li, Shuo Wang, Chao Wang, Fangfang Huang, Qi Gao, Lijuan Wu, Lixin Tao, Jin Guo, Wei Wang, Xiuhua Guo

**Affiliations:** 1 Department of Epidemiology and Health Statistics, School of Public Health, Capital Medical University, Beijing, China; 2 Beijing Municipal Key Laboratory of Clinical Epidemiology, Beijing, China; 3 Graduate Entry Medical School, University of Limerick, Limerick, Ireland; 4 Beijing Chaoyang Hospital, Capital Medical University, Beijing, China; 5 School of Medical Sciences, Edith Cowan University, Perth, WA, Australia; The Ohio State University, UNITED STATES

## Abstract

**Background:**

Heavy fine particulate matter (PM_2.5_) air pollution occurs frequently in China. However, epidemiological research on the association between short-term exposure to PM_2.5_ pollution and respiratory disease morbidity is still limited. This study aimed to explore the association between PM_2.5_ pollution and hospital emergency room visits (ERV) for total and cause-specific respiratory diseases in urban areas in Beijing.

**Methods:**

Daily counts of respiratory ERV from Jan 1 to Dec 31, 2013, were obtained from ten general hospitals located in urban areas in Beijing. Concurrently, data on PM_2.5_ were collected from the Beijing Environmental Protection Bureau, including 17 ambient air quality monitoring stations. A generalized-additive model was used to explore the respiratory effects of PM_2.5_, after controlling for confounding variables. Subgroup analyses were also conducted by age and gender.

**Results:**

A total of 92,464 respiratory emergency visits were recorded during the study period. The mean daily PM_2.5_ concentration was 102.1±73.6 μg/m^3^. Every 10 μg/m^3^ increase in PM_2.5_ concentration at lag_0_ was associated with an increase in ERV, as follows: 0.23% for total respiratory disease (*95%* confidence interval *[CI]*: 0.11%-0.34%), 0.19% for upper respiratory tract infection (URTI) (*95%CI*: 0.04%-0.35%), 0.34% for lower respiratory tract infection (LRTI) (*95%CI*: 0.14%-0.53%) and 1.46% for acute exacerbation of chronic obstructive pulmonary disease (AECOPD) (*95%CI*: 0.13%-2.79%). The strongest association was identified between AECOPD and PM_2.5_ concentration at lag_0-3_ (3.15%, *95%CI*: 1.39%-4.91%). The estimated effects were robust after adjusting for SO_2_, O_3_, CO and NO_2_. Females and people 60 years of age and older demonstrated a higher risk of respiratory disease after PM_2.5_ exposure.

**Conclusion:**

PM_2.5_ was significantly associated with respiratory ERV, particularly for URTI, LRTI and AECOPD in Beijing. The susceptibility to PM_2.5_ pollution varied by gender and age.

## Introduction

Because of the rapid industrialization and economic growth experienced over the past ten years, air pollution has become a serious issue in China. Heavy haze-fog episodes have frequently occurred in recent years [1, 2]. From 2013 to 2014, the annual mean fine particulate matter (PM_2.5_, particles with an aerodynamic diameter ≤ 2.5 μm) concentration for most of the 31 provincial capital cities in China exceeded the Chinese Ambient Air Quality Standards Grade II standard (35 μg/m^3^) [3]. In particular, as the capital of China, Beijing has experienced a more serious challenge in terms of deteriorating air quality because of the coal burning and wind-blown dust from the surrounding industrial cities as well as the vehicle exhaust from the rapidly growing number of automobiles [4]. For example, starting in early January 2013, Beijing experienced multiple prolonged periods of severe smog; the peak hourly concentration of ambient PM_2.5_ soared to 800 μg/m^3^, and the annual ambient PM_2.5_ concentration in 2013 reached 89.5 μg/m^3^ [5]. The extremely high ambient PM_2.5_ concentration has attracted extensive public attention because of the adverse health effects.

Toxicological evidence suggests that the exposure of PM_2.5_ can cause lung inflammation and affect pulmonary immune function [6]. During the past two decades, several studies have demonstrated the effects of PM_2.5_ on the mortality for total respiratory disease [5, 7] or on the number of hospital admissions due to cause-specific respiratory diseases, such as pneumonia, chronic obstructive pulmonary disease and asthma [8–11]. However, few reports have directly evaluated the effects of PM_2.5_ on total and cause-specific respiratory diseases using hospital emergency room visits (ERV) as a morbidity indicator. In addition, most of these studies were conducted in Western developed countries; however, because of the different level of effects, such as ambient PM_2.5_ levels, characteristics of ambient PM_2.5_, population susceptibility, and weather patterns, there is still a need to assess the health effects of PM_2.5_ exposure in developing countries such as China.

It was not until March 1, 2012, that the PM_2.5_ level was required to be included in the air quality standards in China [1]. Previous studies evaluating the short-term adverse effects of ambient particles pollution in China have primarily focused on the effects of PM_10_ (particles with an aerodynamic diameter ≤ 10 μm) [12, 13]. Researchers have suggested that PM_2.5_ is more harmful to health than PM_10_ is, because smaller particles are more readily able to deposit deeper into the lungs and can carry larger concentrations of adsorbed or condensed toxic air pollutants per unit mass. Although numerous studies have reported the effects of PM_2.5_ on mortality [14, 15], a recent meta-analysis has noted that the observed association between ambient PM_2.5_ and morbidity in China has not been validated [16]. Although one study reported the association between PM_2.5_ and hospital admissions for respiratory diseases in Beijing, the correlation was explored from the perspective of geography and spatial analysis using grey correlation analysis, and it considered particles measuring less than 1.0 μm [17]. Another study employed hospital admissions data from one hospital in the Haidian district in Beijing, but it did not detect the effects of PM_2.5_ on cause-specific respiratory diseases [18].

The availability of the thorough monitoring network for PM_2.5_ from Beijing Environmental Protection Bureau since October 2012 provided us with an opportunity to assess the adverse effects of PM_2.5_ pollution. This study aimed to explore association between PM_2.5_ concentration and ERV for total and cause-specific respiratory diseases using a time-series design by controlling for long-term trends and seasonality in daily ERV, day of the week, and weather variables in Beijing. Subgroup analyses were also performed using different age groups and gender to identify the most susceptible subpopulations.

## Materials and Methods

### Ethics statement

The study protocol was approved by the Institutional Review Board at the School of Public Health, Capital Medical University. Written informed consent was not required because we used aggregated data and no individualized data. The patient information was anonymized and de-identified prior to analysis.

### Study site

Beijing is located in the north of China (latitude 39°54'N and longitude 116°25'E). As the capital, Beijing is the political, economical and cultural center of China, with six urban districts (Dongcheng, Xicheng, Chaoyang, Haidian, Fengtai, Shijingshan) and ten suburban districts (Changping, Mentougou, Daxing, Fangshan, Tongzhou, Shunyi, Huairou, Pinggu, Minyun and Yanqing). According to the Chinese Health Statistical Yearbook, in 2012, Beijing had a population of 20,690,000, with 10,685,164 males (51.64%) and 10,004,836 females (48.36%). Approximately 75% of Beijing's total population live in six urban districts, which cover all high population density areas in Beijing (> 5,000 people/km^2^) [19].

### Data collection

**Fig 1:**
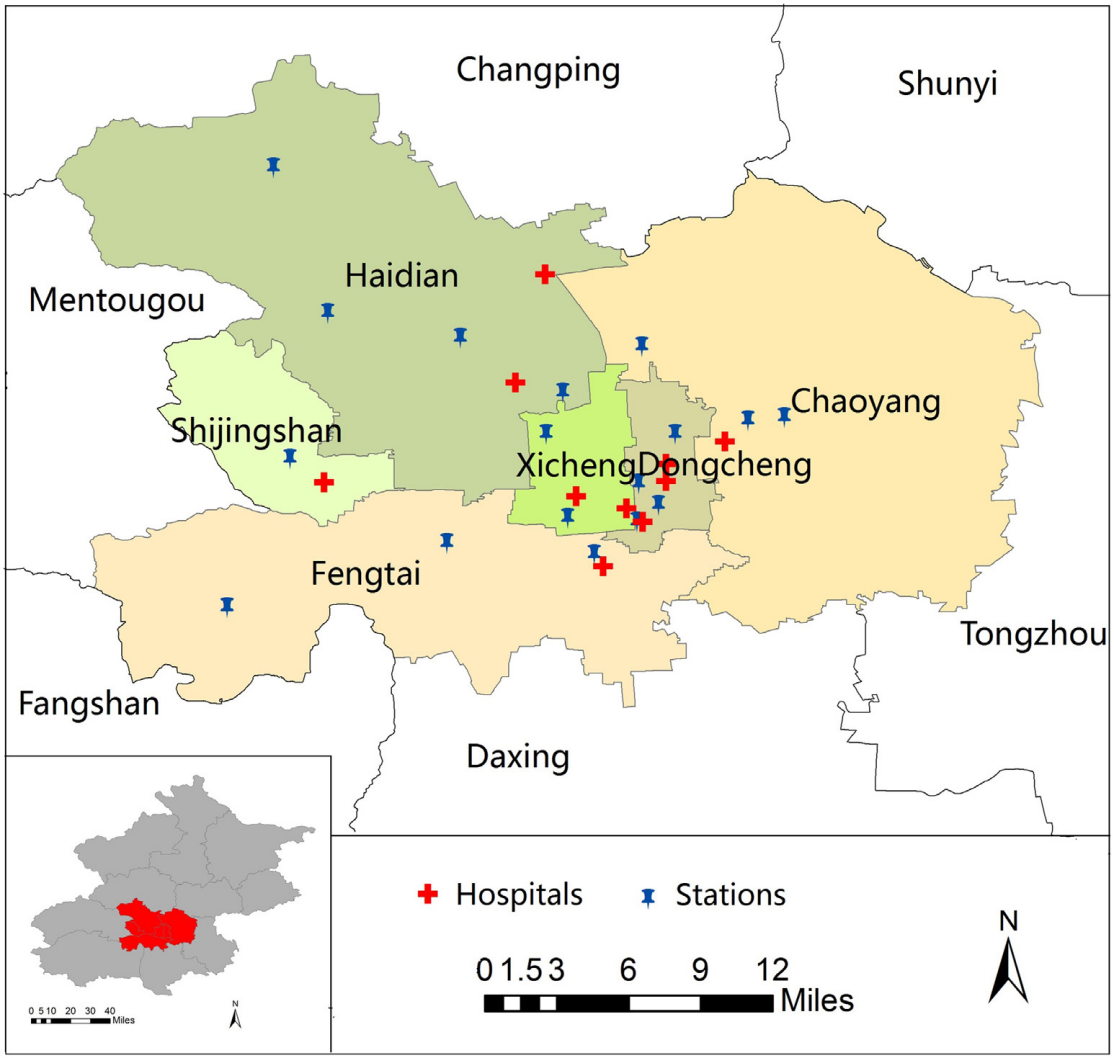
The distribution of 17 monitoring stations for air pollutants and 10 hospitals located in urban areas in Beijing, China. Note: ArcGIS (ArcMap, version 10.0, ESRI Inc. Redlands, CA, USA) was used to create the map.

Daily data on emergency room visits from Jan 1 to Dec 31, 2013, were collected from ten general hospitals located in urban areas in Beijing. To obtain a representative sample of the urban population, one to three publicly funded hospitals in each urban district were selected (Fig 1). Patient data were derived from the computerized medical record system including age, gender, visit date and diagnosis at discharge. ERV for respiratory diseases were extracted according to the principal diagnosis, using the International Classification of Diseases-(ICD) 10th Revision Code of J00-J99. Specifically, we focused on total respiratory disease (ICD-10: J00-J99), upper respiratory tract infection (URTI) (ICD-10: J00, J02-J06), lower respiratory tract infection (LRTI) (ICD-10: J12-J22, J44 [except J44.103 and J44.901]), acute exacerbation of chronic obstructive pulmonary disease (AECOPD) (ICD-10: J44.103, J44.901) and asthma (ICD-10: J45-46).

Since October 2012, daily average and hourly real-time air pollution data were released to the public through a web platform (http://zx.bjmemc.com.cn/) by Beijing Environmental Protection Bureau, which established 35 ambient air quality monitoring stations in the 16 districts of Beijing city. Daily 24-hour average concentration data for PM_2.5_ (unit, μg/m^3^), sulfur dioxide (SO_2_) (unit, μg/m^3^), ozone (O_3_) (unit, μg/m^3^), nitrogen dioxide (NO_2_) (unit, μg/m^3^) and carbon monoxide (CO) (unit, mg/m^3^) levels during the study period were collected, including data from 17 ambient air quality monitoring stations located in urban areas in Beijing city. (Fig 1). The rates of the missing values from the 17 monitoring stations ranged from 9.0% to 15.6%. Some daily data were missing for all districts, primarily due to technical problems (i.e., website maintenance) during the study period. Imputation will produce errors, therefore, we did not fill in the missing values. We used the mean PM_2.5_ concentrations from the 17 monitoring stations to represent the daily exposure of the urban population. In addition, to allow for the effects of weather conditions, during the same period, we obtained daily meteorological data from the Chinese Meteorological Bureau, including the mean temperature and relative humidity.

### Data analysis

A generalized-additive model (GAM) with the Poisson link function was used to explore the association between the daily PM_2.5_ concentration and outcomes, after adjusting for temperature, relative humidity, day of the week (*DOW*), public holidays and influenza outbreaks. First, the basic model was set up, excluding air pollutant variables. The natural cubic smoothing spline (*ns*) functions of calendar time, daily mean temperature and relative humidity were incorporated in the model, allowing for nonlinear relationships with the outcomes. The partial autocorrelation function was used to guide the selection of the best degrees of freedom (*df*) for the spline function of calendar time. Note that the absolute values of the partial autocorrelation function for the model residuals had to be < 0.1 for the first 2 lag days [20], and then the Akaike Information Criterion (*AIC*) was used to determine the best *df* for calendar time. Thus, a natural cubic smoothing spline for calendar time (*df* = 11) was used to control for the seasonal and long-term trends. Natural cubic smoothing spline for temperature and relative humidity (*df* = 3) on current day of ERV occurred was incorporated into the model, based on previous studies [21, 22]. The day of the week (*DOW*), public holidays and influenza outbreaks were all categorical variables. After the basic model was established, the air pollutant variables were then added. The final model is described below:

$$\begin{array}{l} Log\left[E\left(Y_{t}\right)\right]\;=\;\alpha \;+\;\beta_{1}PM2.5_{t-i}\;+\;ns\left(Temp_{0},\;df\;=\;3\right)\;+\;ns\left(RH_{0},\;df\;=\;3\right)\\ \;+\;ns\left(Time,\;df\;=\;11\right)\;+\;\beta_{2}DOW\;+\;\beta_{3}Holiday\;+\;\beta_{4}Influenza \end{array}$$

*E(Y*_*t*_*)* is the expected count for respiratory ERV on day *t*; *PM2*.*5*_*t-i*_ is the mean PM_2.5_ concentration from 17 monitoring stations on day *t*, and *i* is the day lag; ns is the natural cubic splines; *Temp*_*0*_ and *RH*_*0*_ are the daily mean temperature (*°C*) and relative humidity (*%*) of the current day, respectively; *Time* denotes long-term trends and seasonality using the calendar time days; *DOW* is the day of the week; *holiday* indicates a public holiday on day *t* (0 indicates no holiday, and 1 indicates a holiday); *influenza* is a dummy variable for the weeks, with a number of influenza ERV exceeding the 75 percentile in a year [23].

Smoothing function in GAM was used to graphically analyze the exposure-response relationship to verify the assumption of linearity between the predicted log-relative risk of respiratory ERV and PM_2.5_ concentration. The linear effects of PM_2.5_ were then estimated for the current day and up to 5 day before the outcome (lag_0_ to lag_5_). Considering that a single-day lag model might underestimate the association [19], the overall cumulative effects were estimated using 2-day, 4-day and 6-day moving averages of PM_2.5_ concentrations (lag_0-1_, lag_0-3_ and lag_0-5_). We also investigated whether the associations were still sensitive after adjusting for the other gaseous pollutants (SO_2_, O_3_, CO or NO_2_) in two-pollutant models [24]. In the single-pollutant models, PM_2.5_ was placed in the model alone; in the two-pollutant models, SO_2_, O_3_, CO or NO_2_ was jointly included with PM_2.5_. Effects across age groups (0–14 years, 15–34 years, 35–59 years, and ≥ 60 years) and genders were examined using the respiratory ERV subgroups for the health outcomes to identify the most susceptible subpopulation [25]. A Z-test was then used to test the statistical significance of differences by gender or age by calculating $(\beta 1\;-\;\beta 2)/\sqrt{SE_{1}^{2}\;+\;SE_{2}^{2}}$, where *β*_*1*_ and *β*_*2*_ are the estimates coefficient for the two categories (i.e., male and female patients), and *SE*_*1*_ and *SE*_*2*_ are the respective standard errors [26].

Sensitivity analyses were conducted to examine the impact of PM_2.5_ on total respiratory ERV using different *df*: time trend (6–12), temperature (2–6) and relative humidity (2–6). Studies have shown that temperature effects on health would lag for more than 10 days [27]; therefore, the effects of PM_2.5_ were also checked in our model using the 14-day moving average temperature [28]. All sensitivity analyses were performed only for total respiratory ERV in the whole population.

All analyses were performed using the “mgcv” and “nlme” packages in the statistical environment R 3.2.2 (Development Core Team, 2015). The results are presented as the percentage change (PC) and 95% confidence interval (*CI*) in the daily respiratory ERV per 10 μg/m^3^ increase in PM_2.5_ concentration.

## Results

### Descriptive statistics

A total of 92,464 emergency visits for total respiratory diseases were recorded from the ten hospitals in 2013, with URTI accounting for the largest proportion (55.3%). The mean daily count for total respiratory disease was 253 (range, 129 to 556), and 45.5% were male patients. The mean patient age was 44.1 years for male patients and 43.0 years for female patients. There were 19 patients (0.021%) with missing gender information and 6 patients (0.006%) with missing age information, which had little effect on the subgroup analyses. The mean daily visits for cause-specific respiratory diseases were URTI (113 visits), LRTI (122 visits), AECOPD (2 visits) and asthma (6 visits) (Table 1).

**Table 1:** Characteristics and distribution of the main health outcomes and sub-categories of the different gender and age groups.

	Total respiratory disease		URTI		LRTI		AECOPD		Asthma	
	N	Daily Median (P_25_–P_75_)	N	Daily Median (P_25_–P_75_)	N	Daily Median (P_25_–P_75_)	N	Daily Median (P_25_–P_75_)	N	Daily Median (P_25_–P_75_)
Total	92464	241 (207–275)	51134	126 (108–155)	34904	93 (76–109)	712	2 (1–3)	2052	5 (4–7)
Gender										
Male	42095	110 (96–127)	22119	55 (47–68)	16877	45 (37–54)	425	1 (0–2)	867	2 (1–3)
Female	50350	130 (109–151)	29003	71 (60–89)	18021	47 (38–57)	287	1 (0–1)	1185	3 (2–4)
Age group										
0–14	5159	13 (9–18)	3002	8 (5–11)	2030	5 (3–7)	0	0 (0–0)	53	0 (0–0)
15–34	38228	99 (83–117)	28304	72 (59–87)	7973	20 (15–28)	1	0 (0–0)	431	1 (0–2)
35–59	24792	63 (54–76)	14111	35 (28–44)	8471	22 (17–28)	79	0 (0–0)	868	2 (1–3)
60-	24279	65 (54–74)	5713	14 (10–19)	16428	44 (37–51)	632	2 (1–3)	700	2 (1–3)

Note: URTI-upper respiratory tract infection, LRTI-lower respiratory tract infection, AECOPD-acute exacerbation of chronic obstructive pulmonary disease, N-counts of event, daily median (P_25_–P_75_)-median (25^th^ percentile and 75^th^ percentile) of daily event counts.

**Fig 2:**
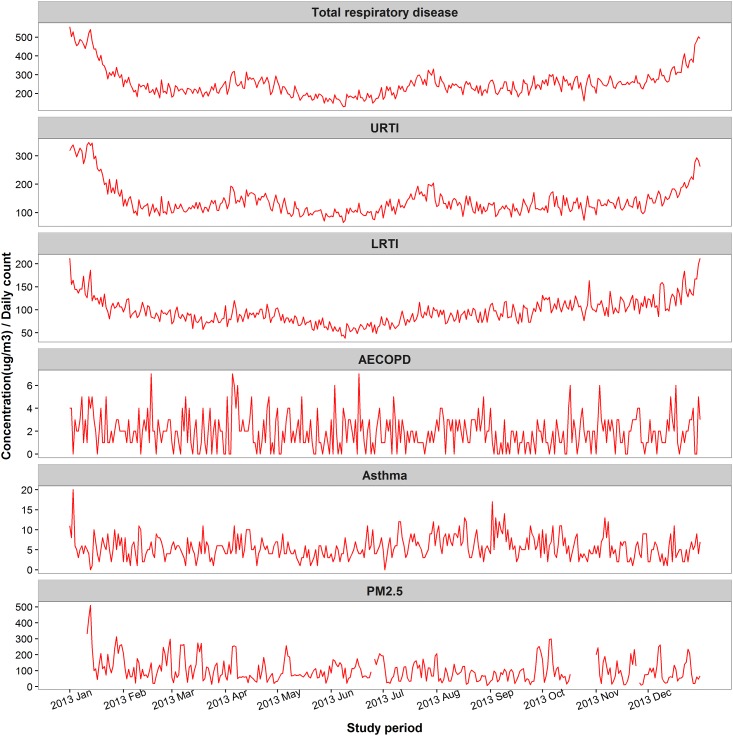
Time series plot of emergency room visits for respiratory diseases (number of daily cases) and PM_2.5_ concentrations in Beijing, China during study period. Abbreviations: URTI-upper respiratory tract infection, LRTI-lower respiratory tract infection, AECOPD-acute exacerbation of chronic obstructive pulmonary disease, PM_2.5_- particles with an aerodynamic diameter ≤ 2.5 μm.

During the study period, the overall mean daily PM_2.5_ concentration was 102.1 μg/m^3^ (range, 6.7 μg/m^3^ to 508.5 μg/m^3^). In terms of the Chinese Ambient Air Quality Standards Grade II standards for 24-hour average PM_2.5_ concentration (≤ 75 μg/m^3^), 45.7% (155 days) of the daily PM_2.5_ concentrations were below this standard during the study period. However, in terms of the WHO Air Quality Standards (24-hour average PM_2.5_ concentration ≤ 25 μg/m^3^), only 8.8% (30 days) days met the standard. The mean daily concentrations of SO_2_, O_3_, CO and NO_2_ were 31.2 μg/m^3^, 104.3 μg/m^3^, 2.2 mg/m^3^ and 66.7 μg/m^3^, respectively (Table 2). All Spearman correlations among the pollutants and weather variables were statistically significant at 0.05 level (2-tailed). Generally, PM_2.5_ was strongly correlated with SO_2_, CO and NO_2_ (*r*_*s*_ = 0.56, 0.80 and 0.72, respectively) and negatively correlated with O_3_ (*r*_*s*_ = –0.15). The time series graph showed the daily variations of ERV for respiratory diseases and PM_2.5_ concentrations during study period (Fig 2).

**Table 2:** Summary of the environmental and meteorological variables in urban areas in Beijing during the study period.

Variables			Percentiles				
	Mean	SD	Min	25^th^	Median	75^th^	Max
PM_2.5_ (μg/m^3^)	102.1	73.6	6.7	53.1	82.4	129.5	508.5
SO_2_ (μg/m^3^)	31.2	25.7	3.6	11.0	23.3	43.7	133.8
O_3_ (μg/m^3^)	104.3	68.3	4.4	53.0	89.4	146.5	305.4
CO (mg/m^3^)	2.2	1.5	0.3	1.2	1.9	2.6	17.7
NO_2_ (μg/m^3^)	66.7	25.0	12.4	50.1	62.3	78.3	171.5
Temperature (°C)	11.3	11.6	-12.6	1.2	11.0	22.2	29.0
Relative humidity (%)	58.7	17.3	18.9	46.7	59.0	73.3	93.3

Abbreviations: PM_2.5_-particles with an aerodynamic diameter ≤ 2.5 μm, SO_2_-sulfur dioxide, O_3_-ozone, CO-carbon monoxide, NO_2_-nitrogen dioxide, SD-standard deviation, Max-maximum, and Min-minimum.

### Associations between PM_2.5_ and ERV for respiratory disease

**Fig 3:**
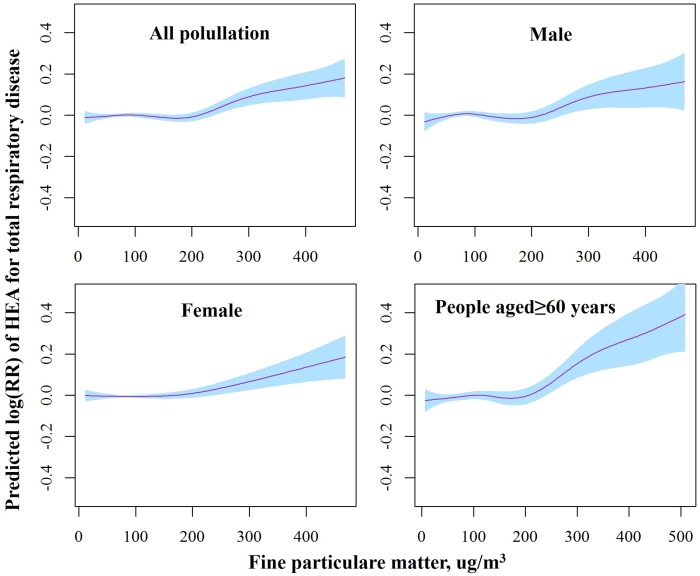
The smoothed exposure-response curves of daily average PM_2.5_ concentrations at lag_0-1_ against the risk of total respiratory ERV in different subgroups. Note: The X-axis shows the moving averages of PM_2.5_ concentrations at lag_0-1_ (μg/m^3^). The Y-axis is the predicted log relative risk (*RR*). The pink line represents central estimates, and the blue shaded area represents the *95%* CI.

There were clear exposure-response relationships between PM_2.5_ concentration and total respiratory ERV (Fig 3). The exposure-response relationships were approximately linear, with a tiny fluctuation when the PM_2.5_ concentrations were below 200 μg/m^3^ and a sharper response at higher PM_2.5_ concentrations.

**Fig 4:**
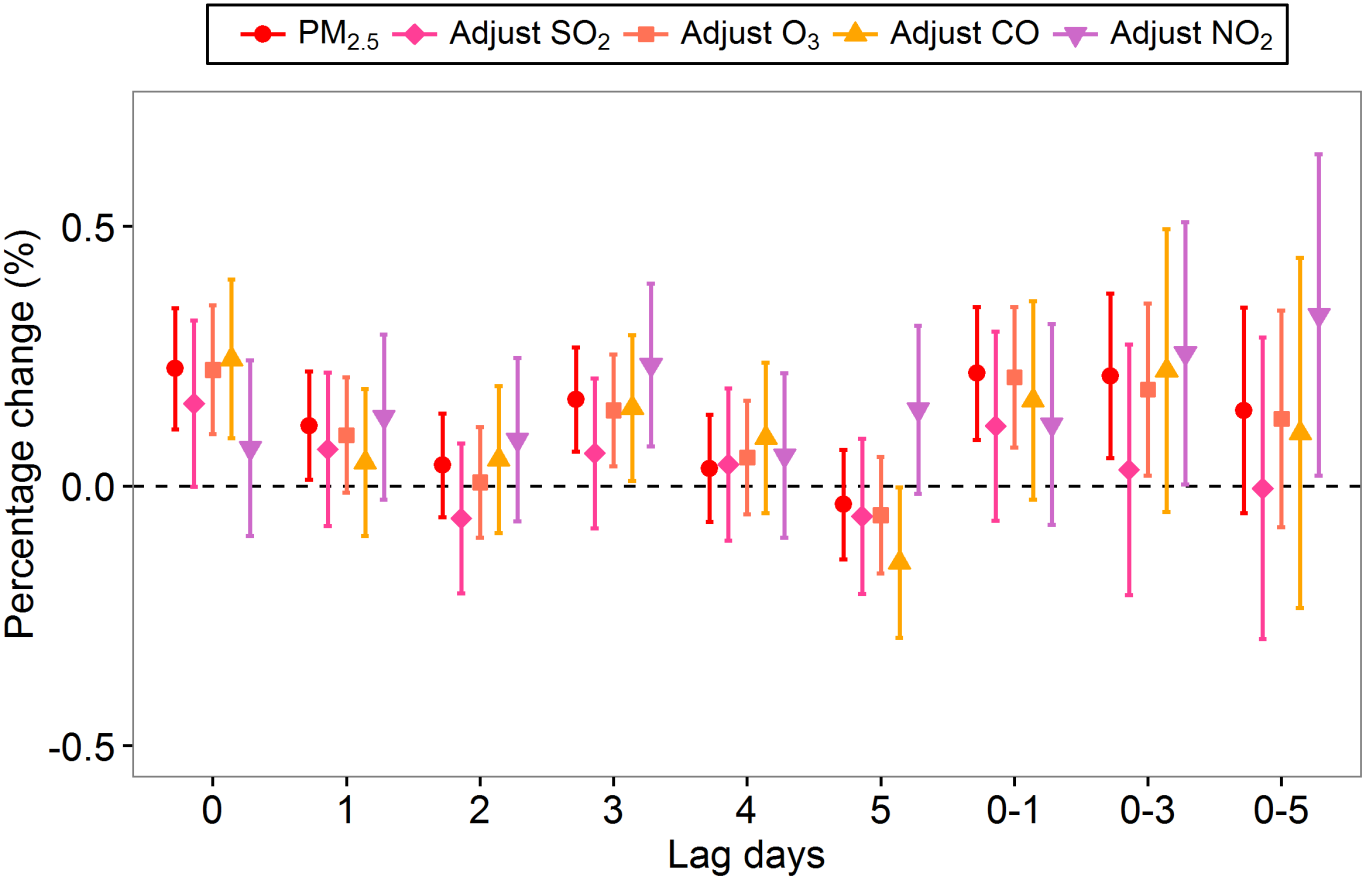
Percentage increase of total respiratory ERV associated with a 10 μg/m^3^ increase in PM_2.5_ concentrations using the single- and two-pollutant models.

Fig 4 shows the associations between the PM_2.5_ concentration and total respiratory ERV. We observed statistically significant associations between the total respiratory ERV and PM_2.5_ concentration on the current day (lag_0_), the previous day (lag_1_), the previous three day (lag_3_), the 2-day moving average (lag_0-1_) and the 4-day moving average (lag_0-3_). We estimated increases of 0.23% (*95%CI*: 0.11%-0.34%), 0.12% (*95%CI*: 0.01%-0.22%), 0.17% (*95%CI*: 0.07%-0.27%), 0.22% (*95%CI*: 0.09%-0.35%) and 0.21% (*95%CI*: 0.05%-0.37%) in total respiratory ERV associated with every 10 μg/m^3^ increase in PM_2.5_ at lag_0_, lag_1_, lag_3_, lag_0-1_ and lag_0-3_, respectively. The largest estimated effect was observed on the current day (lag_0_). The estimated effects of PM_2.5_ on total respiratory ERV changed little after adjusting for O_3_, but fluctuated with attenuating tendencies after adjusting for SO_2_ or CO and increasing tendencies after adjusting for NO_2_. Significant results were observed at different lag days in the two-pollutant models (Fig 4).

Table 3 lists the effects of PM_2.5_ on cause-specific respiratory ERV. Every 10 μg/m^3^ increase of PM_2.5_ on the current day (lag_0_) was associated with an increase in ERV: 0.19% for URTI (95%*CI*: 0.04%-0.35%), 0.34% for LRTI (95%*CI*: 0.34%-0.53%), and 1.46% for AECOPD (95%*CI*: 0.13%-2.79%), respectively. Single-day delayed effects of PM_2.5_ were significant with URTI on lag_1_ and lag_3_, with LRTI on lag_2_ and lag_3_, and with AECOPD on lag_1_ and lag_2_. For the cumulative effects, the 2-day moving average PM_2.5_ concentration (lag_0-1_) was significantly associated with ERV for URTI, LRTI and AECOPD. The estimated effect of PM_2.5_ on AECOPD at lag_0-3_ (3.15%, *95%CI*: 1.39%-4.91%) was the largest. No significant association was found between PM_2.5_ and asthma ERV at any lag patterns (P>0.05). The estimated effects of PM_2.5_ on cause-specific respiratory ERV in two-pollutant models are shown in the supporting information, S1 Table.

**Table 3:** Percentage changes with 95% CI in cause-specific respiratory ERV associated with a 10 μg/m^3^ increase in PM_2.5_ concentrations for different lag structures.*

Lag Days	URTI		LRTI		AECOPD		Asthma	
	PC	95% CI	PC	95% CI	PC	95% CI	PC	95% CI
lag_0_	**0.19**	**(0.04, 0.35)**	**0.34**	**(0.14, 0.53)**	**1.46**	**(0.13, 2.79)**	-0.62	(-1.46, 0.22)
lag_1_	**0.14**	**(0.00, 0.27)**	0.08	(-0.09, 0.25)	**2.40**	**(1.26, 3.53)**	0.19	(-0.54, 0.93)
lag_2_	-0.09	(-0.22, 0.05)	**0.19**	**(0.03, 0.36)**	**1.22**	**(0.11, 2.34)**	0.07	(-0.63, 0.77)
lag_3_	**0.14**	**(0.00, 0.27)**	**0.19**	**(0.02, 0.35)**	0.92	(-0.22, 2.07)	0.01	(-0.68, 0.70)
lag_4_	0.02	(-0.12, 0.16)	0.09	(-0.08, 0.26)	-0.81	(-2.04, 0.42)	-0.42	(-1.13, 0.29)
lag_5_	0.02	(-0.12, 0.16)	-0.10	(-0.27, 0.08)	-1.48	(-2.75, -0.22)	-0.33	(-1.05, 0.39)
lag_0-1_	**0.20**	**(0.03, 0.37)**	**0.27**	**(0.06, 0.48)**	**2.70**	**(1.25, 4.14)**	-0.21	(-1.13, 0.70)
lag_0-3_	0.18	(-0.03, 0.39)	0.24	(-0.02, 0.50)	**3.15**	**(1.39, 4.91)**	0.09	(-1.00, 1.19)
lag_0-5_	0.10	(-0.17, 0.37)	0.21	(-0.11, 0.53)	1.81	(-0.43, 4.06)	-0.36	(-1.70, 0.99)

*statistically significant results at the 5% level are indicated in bold.

Abbreviations: URTI-upper respiratory tract infection, LRTI-lower respiratory tract infection, AECOPD-acute exacerbation of chronic obstructive pulmonary disease, PC- percentage change.

**Fig 5:**
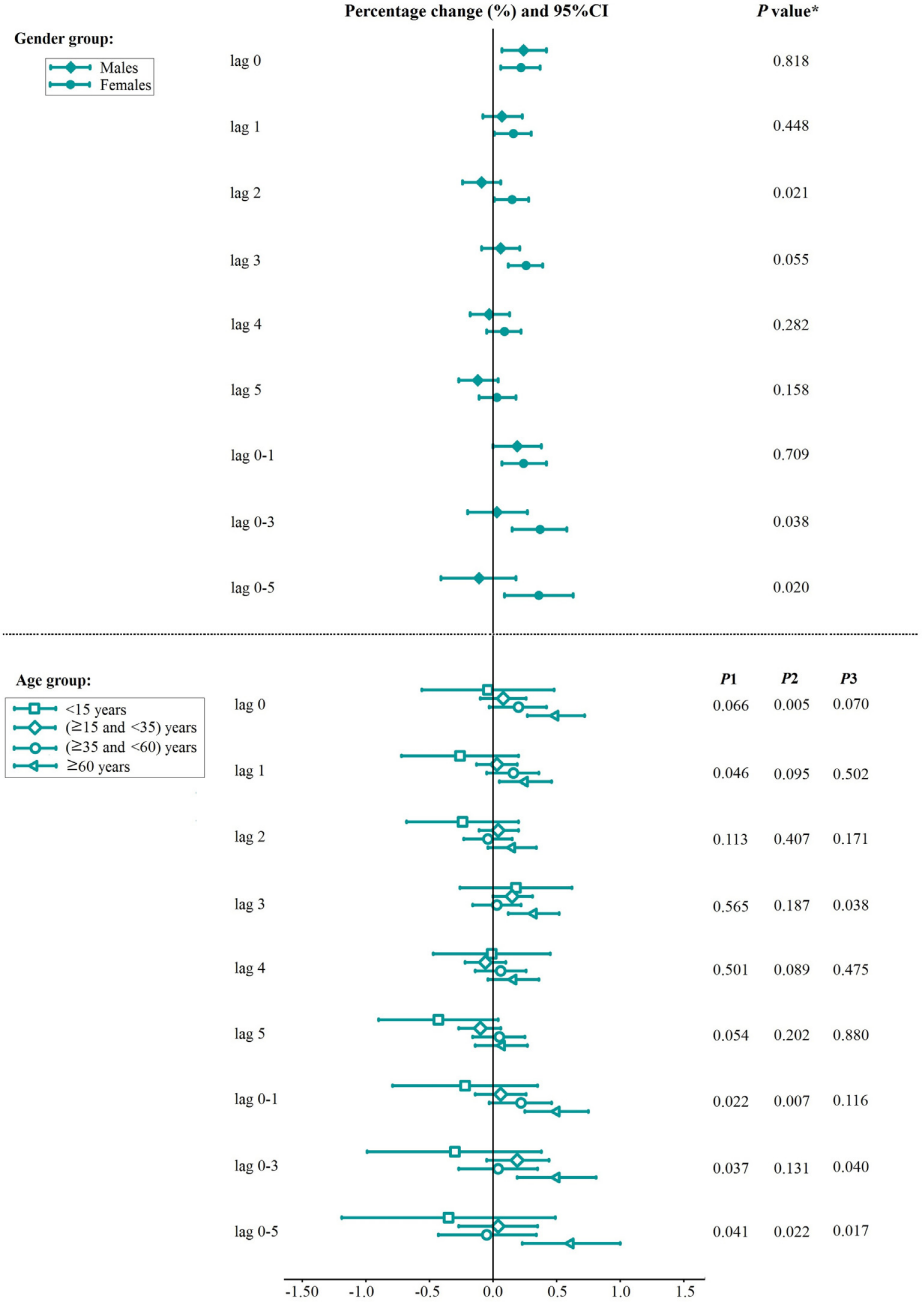
Percentage change with 95%CI in total respiratory ERV associated with a 10 μg/m^3^ increase in PM_2.5_ concentrations by gender and age. Note: The *P* Value obtained from the Z-test for the difference between the two relative risks derived from the subgroup analysis. *P*_*1*_: 60 years of age and older vs the age group younger than 15 years; *P*_*2*_: 60 years of age and older vs the age group 15 years of age and older and younger than 35 years; *P*_*3*_: age 60 years of age and older vs the age group 35 years of age and older and younger than 60 years.

The association between PM_2.5_ and total respiratory ERV differed by gender and age group (Fig 5). PM_2.5_ exposure resulted in slightly larger effects in females than in males at all lag patterns. Meanwhile, the gender differences were statistically significant at lag_2_, lag_0-3_ and lag_0-5_ (*P*<0.05). We observed that the estimated effects of PM_2.5_ on total respiratory ERV among people 60 years of age and older were significant at all lag patterns and higher when compared with other age groups. In terms of the estimated effects, the differences between age groups were statistically significant in the following categories: between people 60 years of age and older and the age group younger than 15 years at lag_0-1_, lag_0-3_ and lag_0-5_; between people 60 years of age and older and the age group 15 years of age and older and younger than 35 years at lag_0_, lag_0-1_ and lag_0-5_; and between people 60 years of age and older and the age group 35 years of age and older and younger than 60 years at lag_0_, lag_3_, lag_0-3_ and lag_0-5_ (*P*<0.05) (Fig 5).

### Sensitivity analysis results

S2 Table shows the percentage change in total respiratory ERV associated with a 10 μg/m^3^ increase in PM_2.5_ on the current day (lag_0_), under different *df* for calendar time, temperature and relative humidity. The *AIC* of the basic model was lowest, and the estimated effect became stable when *df* of spline for calendar time was 11; different *df* of spline for temperature and relative humidity had little effect on the estimated effect, suggesting that the findings on the associations of PM_2.5_ concentration with respiratory ERV were robust in our study. The estimated effects changed little when the 14-day moving average temperature was controlled for in our model. As shown in S3 Table, similar results have been found.

## Discussion

This study provided strong evidence of the associations between PM_2.5_ and respiratory disease morbidity in Beijing. PM_2.5_ was significantly associated with ERV for total respiratory disease and cause-specific respiratory diseases, including URTI, LRTI, and AECOPD. Females and people 60 years of age and older suffered a higher risk from PM_2.5_ exposure. To our knowledge, this study is one of the few that have thoroughly investigated the short-term effects of PM_2.5_ air pollution on total and cause-specific respiratory diseases.

Previous studies have identified significant positive associations between PM_2.5_ and respiratory morbidity in Beijing, but they were restricted to one or two districts [17, 18]. To obtain more representative results for the population in the entire city, we correlated the daily ERV for respiratory diseases from ten hospitals located in the six urban districts in Beijing with the citywide daily mean PM_2.5_ concentration from 17 monitoring stations. We were not able to allot each patient a unique PM_2.5_ concentration because we did not have individual’ information on his or her residential address. We assumed that residents would be more likely to go to the nearest hospital in Beijing, therefore, we assigned the monitored concentrations to patients based on their proximity to the hospitals, and then we used generalized additive mixed model (GAMM) to examine the impacts of the spatially assigned PM_2.5_ concentration on health outcomes. However, the estimated effects using spatially assigned PM_2.5_ concentrations in the GAMM were slightly smaller, and the confidence intervals were larger than those using mean PM_2.5_ in the GAM (supporting information, S1 Fig and S4 Table), which was not consistent with the results reported by Xu M, et al [21], likely due to the larger standard error generated by more missing values of air pollutants in the GAMM in our study or because of the smaller spatial variation of PM_2.5_ concentrations in urban areas compared to that in the entire city.

Toxicological studies have proposed that acute impairment of the lung cellular defense and immune system might be causative mechanisms for the short-term effects of PM_2.5_ on respiratory disease [29]. Our current findings on the short-term associations between PM_2.5_ and respiratory ERV are consistent with other toxicological and epidemiological findings. To date, many epidemiological studies have examined and reported the adverse health effects of PM_2.5_ on hospital admissions for LRTI, COPD and asthma [8–11]; however, few reports have directly addressed total respiratory disease using ERV as a morbidity indicator. Our study found statistically significant associations between PM_2.5_ and ERV for total respiratory disease and cause-specific diseases, including URTI, LRTI and AECOPD. Likely due to the higher PM_2.5_ concentration in Beijing in 2013, a longer lag association was observed in our study compared to previous reports of a 0–2 day lag [22, 30]. We found that the effects of PM_2.5_ on LRTI were higher overall compared to the effects on URTI in our study. LRTI, including pneumonia and acute bronchitis, is more severe and can cause a higher disease burden during an individual’s life span than URTI; therefore, most previous studies likely focused on the effect of PM_2.5_ on LRTI rather than URTI [31, 32]. A study conducted in Rome showed a significant association between PM_2.5_ and LRTI at lag_2_, with a higher effect (2.82%, 95%*CI*: 0.52%-5.19%) than the estimated effect in our study (0.19%, 95%*CI*: 0.09%-0.36%) [30]. Studies of the effects of PM_2.5_ on COPD admissions are rare and inconsistent [9, 31, 32]. We found that the effects of PM_2.5_ on AECOPD are the largest compared with other respiratory diseases groups. Due to the slow progression and chronic nature of the disease, COPD can also result in a massive and growing disease burden that affects a patient’s health quality. Thus, COPD patients should stay at home as long as possible during a PM_2.5_ pollution outbreak to avoid possible acute exacerbation. We did not find any statistically significant associations between PM_2.5_ and asthma ERV, likely due to the small daily number of asthma patients. A simulation study suggested that the power of a time-series study of the acute health effects of air pollution can be increased by increasing either the mean daily count of the outcome or the time-series length [33]. In our study, the total cases of AECOPD and asthma were small, which limited our calculation power. In addition, air pollution health effects over a short time interval (e.g., 1-year time series study) are often small; and lower estimated effects also decrease power [33]. The lack of power may inhibit the identification of an association with specific causes of respiratory diseases that have low counts, as is the case with asthma, especially when the estimated effect is low [34].

When examining the associations between PM_2.5_ and total respiratory ERV, we considered the confounding factors of the other gaseous pollutants. The estimated effects of PM_2.5_ on total respiratory disease were affected by the inclusion of SO_2_, CO or NO_2_, but not O_3_ in the two-pollutant models, possibly caused by the strong correlations between PM_2.5_ and SO_2_ (r_s_ = 0.56), PM_2.5_ and CO (r_s_ = 0.80) or PM_2.5_ and NO_2_ (r_s_ = 0.72). In studies that have estimated the adverse effects of PM_2.5_ in multi-pollutant models, positive associations between PM_2.5_ and respiratory causes persisted after adjusting for other gaseous pollutants [8, 24, 35]. In Taiwan, the PM_2.5_ concentration on the current day (lag_0_) increased hospital admissions for COPD on cool days (<25°C), and the risk estimates reduced after adjustment for O_3_, CO or NO_2_, and increased after adjustment for SO_2_ in the model [8]. In a time-series study conducted in New York City, PM_2.5_ was shown to increase emergency department visits for asthma during warm season in single pollutant models, but the risk estimate was attenuated once SO_2_, O_3_, CO or NO_2_ was included in the model [35]. In another time-series study conducted in Hong Kong, the risk estimate of PM_2.5_ on COPD emergency hospital admissions also became unstable after adjusting for CO and NO_2_ [24]. These results may reflect the actual difference in the toxicity of the corresponding air pollutants themselves, but it is unlikely to differentiate the independent effects of each air pollutant in multi-pollutant models [21]. In the present study, PM_2.5_ was positively associated with respiratory causes ERV in both single-pollutant and multi-pollutant models at different lags, reflecting the actual adverse effects of PM_2.5_ on human respiratory system.

The shape of the exposure—response relationship between PM_2.5_ concentration and respiratory disease risk is crucial to determine the pattern of the adverse response. Most studies have assumed linear associations between particulate matter air pollution and health outcomes in epidemiological studies [7, 12, 18, 19, 24]. Schwartz et al explored the exposure—response association between PM_2.5_ and daily deaths by using a hierarchical model in the United States, and found a linear relationship without threshold [36]. This result is support by a cohort study conducted in Europe, which indicated PM_2.5_ was associated with natural-cause mortality, even within concentration ranges well below the present European annual mean limit value (<25 ug/m^3^) [37]. Tian L et al found that in Hong Kong, the exposure-response curve between PM_2.5_ and COPD was almost identical to that noted by Schwartz et al [24]. However, using regression splines to model the exposure-response relationship between PM_2.5_ and ischemic heart disease morbidity in Beijing, Xie W et al showed that the curve was non-linear, with predicted effect values less than zero at lower concentrations (0–90 ug/m^3^), a stable response at intermediate concentrations (90–180 ug/m^3^) and then a relatively shallower response at higher PM_2.5_ concentrations, which is partly consistent with our results [19]. Samoli et al provided evidence for deviation from linearity at lower level of particulate distribution in a multicity study, which estimated the exposure—response relationships between PM_10_ and mortality [38]. They also proposed that the different city-specific relations can be explained partly by factors characterizing the air pollution mix and climate [38]. For example, Wong CM et al found no clear exposure—response relationships in warm seasons between respiratory mortality and PM_10_, but observed a positive exposure—response relationship when PM_10_ concentrations up to 80 μg/m^3^ in the cool seasons [39].

We found that the effects of PM_2.5_ pollution on total respiratory ERV were significantly greater in females than in males. In previous studies, the gender-specific short-term effects of air pollution were inconsistent [25, 40, 41]. Kan H, et al suggested that nonsmokers may be more sensitive to PM_2.5_ exposure [25], whereas the prevalence of smoking in females is lower than that in males in the Chinese population [42]. Moreover, Sunyer, et al suggested that different particles deposition patterns between males and females may partly explain these differences [43]. The age subgroup analysis results suggested that elderly people might be more susceptible to PM_2.5_ exposure. Elderly people may have a weaker immune system and a higher prevalence of chronic respiratory diseases, thus, they are more vulnerable to PM_2.5_ pollution. Generally, in accordance with previous studies [17, 25, 44], our study suggested that special attention should be paid to elderly people, in terms of their PM_2.5_ exposure.

Compared with previous studies focusing on the associations between PM_2.5_ pollution and respiratory diseases in relatively lower polluted areas in developed countries, our study investigated the association between PM_2.5_ pollution and respiratory ERV in a heavy polluted city (i.e., Beijing, China). Given the high level of PM_2.5_ pollution in Beijing, we were able to explore the exposure-response relationship in a wide range of PM_2.5_ concentrations. We used respiratory ERV as the health outcomes, and these unscheduled respiratory diseases therapy were more likely to be community-acquired and might reflect the acute effects of PM_2.5_ pollution. Compared to the other study in Beijing [18], our air pollution data were entirely derived from the 17 monitoring stations in urban areas in Beijing, which were more representative of the general population exposure than data from a single monitoring station.

Several limitations of our study should be mentioned. First, exposure measurement error was inevitable in this time-series study, which has been shown to bias estimates downward [45]. Second, we could not identify the re-admissions for patients with the same respiratory disease from the available data. However, it was possible that some patients, particularly children and elderly people, were admitted to hospitals multiple times in a short time period. Such repeated admissions could lead to temporal dependence among the outcomes, which may lead to an underestimation of the variance of PM_2.5_ pollution risk estimates [46]. Third, we only collected 1 year of emergency data from ten general hospitals in the current study due to the constraints of emergency data availability in multiple hospitals, which might have led to bias in the results and also limit our research power. Therefore, further studies are warranted.

## Conclusions

In conclusion, our study provides evidence that PM_2.5_ pollution can increase the risk of respiratory ERV, particularly for URTI, LRTI and AECOPD in urban areas in Beijing. The strongest association was between AECOPD and the 4-day moving average PM_2.5_ concentration. Different ages and genders varied in their susceptibility to PM_2.5_ pollution. The government should pay more attention to the health effects of PM_2.5_ pollution and provide appropriate solutions to control PM_2.5_ pollution to improve air quality.

## Supporting Information

S1 Database Air pollution data, meteorological data and emergency data. (XLS)

S1 Fig Percentage increase of total respiratory ERV associated with a 10 μg/m^3^ increase in PM_2.5_ concentrations in GAM and GAMM. (TIF)

S1 Table Percentage changes with 95% CI in cause-specific respiratory ERV associated with a 10 μg/m^3^ increase in PM_2.5_ concentrations for the different lag structures in two-pollutant models. * (DOC)

S2 Table Percentage changes with 95% CI in total respiratory ERV associated with a 10 μg/m^3^ increase in the PM_2.5_ concentrations at lag_0_ by different degrees of freedom (*df*) for time, temperature, and relative humidity. (DOC)

S3 Table Percentage changes with 95% CI in total respiratory ERV associated with a 10 μg/m^3^ increase in PM_2.5_ concentrations when the current day’s temperature and 14-day moving average temperature were controlled. (DOC)

S4 Table Percentage changes with 95% CI in cause-specific respiratory ERV associated with a 10 μg/m^3^ increase in PM_2.5_ concentrations for different lag structures in single-pollutant GAMM. * (DOC)

## References

[1] Dominici F, Mittleman MA. China’s air quality dilemma: reconciling economic growth with environmental protection. JAMA. 2012; 307(19): 2100–2. 10.1001/jama.2012.4601 22665112

[2] Li M, Zhang L. Haze in China: current and future challenges. Environ Pollut. 2014; 189: 85–6. 10.1016/j.envpol.2014.02.024 24637256

[3] Wang Y, Ying Q, Hu J, Zhang H. Spatial and temporal variations of six criteria air pollutants in 31 provincial capital cities in China during 2013–2014. Environ Int. 2014; 73: 413–22. 10.1016/j.envint.2014.08.016 25244704

[4] Li L, Liu DJ. Study on an air quality evaluation model for Beijing City under haze-fog pollution based on new ambient air quality standards. Int J Environ Res Public Health. 2014; 11(9): 8909–23. 10.3390/ijerph110908909 25170682 PMC4198997

[5] Zhou M, He G, Fan M, Wang Z, Liu Y, Ma J, et al Smog episodes, fine particulate pollution and mortality in China. Environ Res. 2015; 136: 396–404. 10.1016/j.envres.2014.09.038 25460661

[6] Zhao H, Li W, Gao Y, Li J, Wang H. Exposure to particular matter increases susceptibility to respiratory Staphylococcus aureus infection in rats via reducing pulmonary natural killer cells. Toxicology. 2014; 325: 180–8. 10.1016/j.tox.2014.09.006 25220797

[7] Dai L, Zanobetti A, Koutrakis P, Schwartz JD. Associations of fine particulate matter species with mortality in the United States: a multicity time-series analysis. Environ Health Perspect. 2014; 122(8): 837–42. 10.1289/ehp.1307568 24800826 PMC4123030

[8] Tsai SS, Chiu HF, Liou SH, Yang CY. Short-term effects of fine particulate air pollution on hospital admissions for respiratory diseases: a case-crossover study in a tropical city. J Toxicol Environ Health A. 2014; 77(18): 1091–101. 10.1080/15287394.2014.922388 25072896

[9] Belleudi V, Faustini A, Stafoggia M, Cattani G, Marconi A, Perucci CA, et al Impact of fine and ultrafine particles on emergency hospital admissions for cardiac and respiratory disease. Epidemiology. 2010; 21(3): 414–23. 10.1097/EDE.0b013e3181d5c021 20386174

[10] Tsai SS, Chang CC, Yang CY. Fine particulate air pollution and hospital admissions for chronic obstructive pulmonary disease: a case-crossover study in Taipei. Int J Environ Res Public Health. 2013; 10(11): 6015–26. 10.3390/ijerph10116015 24284359 PMC3863884

[11] Cheng MH, Chen CC, Chiu HF, Yang CY. Fine particulate air pollution and hospital admissions for asthma: a case-crossover study in Taipei. J Toxicol Environ Health A. 2014; 77(18): 1075–83. 10.1080/15287394.2014.922387 25072894

[12] Zheng S, Wang M, Wang S, Tao Y, Shang K. Short-term effects of gaseous pollutants and particulate matter on daily hospital admissions for cardio-cerebrovascular disease in Lanzhou: evidence from a heavily polluted city in China. Int J Environ Res Public Health. 2013; 10(2): 462–77. 10.3390/ijerph10020462 23358231 PMC3635155

[13] Guo Y, Barnett AG, Zhang Y, Tong S, Yu W, Pan X. The short-term effect of air pollution on cardiovascular mortality in Tianjin, China: comparison of time series and case-crossover analyses. Sci Total Environ. 2010; 409(2): 300–6. 10.1016/j.scitotenv.2010.10.013 21055792

[14] Yang C, Peng X, Huang W, Chen R, Xu Z, Chen B, et al A time-stratified case-crossover study of fine particulate matter air pollution and mortality in Guangzhou, China. Int Arch Occup Environ Health. 2012; 85(5): 579–85. 10.1007/s00420-011-0707-7 21960028

[15] Li Y, Ma Z, Zheng C, Shang Y. Ambient temperature enhanced acute cardiovascular-respiratory mortality effects of PM2.5 in Beijing, China. Int J Biometeorol. 2015; 59(12): 1761–70. 10.1007/s00484-015-0984-z 25900003

[16] Lu F, Xu D, Cheng Y, Dong S, Guo C, Jiang X, et al Systematic review and meta-analysis of the adverse health effects of ambient PM_2.5_ and PM_10_ pollution in the Chinese population. Environ Res. 2015; 136: 196–204. 10.1016/j.envres.2014.06.029 25460637

[17] Xiong Q, Zhao W, Gong Z, Zhao W, Tang T. Fine Particulate Matter Pollution and Hospital Admissions for Respiratory Diseases in Beijing, China. Int J Environ Res Public Health. 2015; 12(9): 11880–92. 10.3390/ijerph120911880 26402691 PMC4586713

[18] Li P, Xin J, Wang Y, Wang S, Li G, Pan X, et al The acute effects of fine particles on respiratory mortality and morbidity in Beijing, 2004–2009. Environ Sci Pollut Res Int. 2013; 20(9): 6433–44. 10.1007/s11356-013-1688-8 23589266

[19] Xie W, Li G, Zhao D, Xie X, Wei Z, Wang W, et al Relationship between fine particulate air pollution and ischemic heart disease morbidity and mortality. Heart. 2015; 101(4): 257–63. 10.1136/heartjnl-2014-306165 25341536

[20] Qian Z, Lin HM, Stewart WF, Kong L, Xu F, Zhou D, et al Seasonal pattern of the acute mortality effects of air pollution. J Air Waste Manag Assoc. 2010; 60(4): 481–8. 20437783 10.3155/1047-3289.60.4.481

[21] Xu M, Guo Y, Zhang Y, Westerdahl D, Mo Y, Liang F, et al Spatiotemporal analysis of particulate air pollution and ischemic heart disease mortality in Beijing, China. Environ Health. 2014; 13: 109 10.1186/1476-069X-13-109 25495440 PMC4293109

[22] Dominici F, Peng RD, Bell ML, Pham L, McDermott A, Zeger SL, et al Fine particulate air pollution and hospital admission for cardiovascular and respiratory diseases. JAMA. 2006; 295(10): 1127–34. 16522832 10.1001/jama.295.10.1127 PMC3543154

[23] Qiu H, Yu IT, Wang X, Tian L, Tse LA, Wong TW. Cool and dry weather enhances the effects of air pollution on emergency IHD hospital admissions. Int J Cardiol 2013; 168(1): 500–5. 10.1016/j.ijcard.2012.09.199 23079091

[24] Tian L, Ho KF, Wang T, Qiu H, Pun VC, Chan CS, et al Ambient carbon monoxide and the risk of hospitalization due to chronic obstructive pulmonary disease. Am J Epidemiol. 2014; 180(12): 1159–67. 10.1093/aje/kwu248 25480818

[25] Kan H, London SJ, Chen G, Zhang Y, Song G, Zhao N, et al Season, sex, age, and education as modifiers of the effects of outdoor air pollution on daily mortality in Shanghai, China: The Public Health and Air Pollution in Asia (PAPA) Study. Environ Health Perspect. 2008; 116(9): 1183–8. 10.1289/ehp.10851 18795161 PMC2535620

[26] Altman DG, Bland JM. Interaction revisited: the difference between two estimates. BMJ. 2003; 326(7382): 219 12543843 10.1136/bmj.326.7382.219 PMC1125071

[27] Ma W, Chen R, Kan H. Temperature-related mortality in 17 large Chinese cities: How heat and cold affect mortality in China. Environ Res. 2014; 134: 127–33. 10.1016/j.envres.2014.07.007 25127523

[28] Guo Y, Li S, Tawatsupa B, Punnasiri K, Jaakkola JJ, Williams G. The association between air pollution and mortality in Thailand. Sci Rep. 2014; 4: 5509 10.1038/srep05509 24981315 PMC4076679

[29] Happo MS, Salonen RO, Hälinen AI, Jalava PI, Pennanen AS, Dormans JA, et al Inflammation and tissue damage inmouse lung by single and repeated dosing of urban air coarse and fine particles collected from six European cities. Inhal Toxicol. 2010; 22(5): 402–16. 10.3109/08958370903527908 20121583

[30] Host S, Larrieu S, Pascal L, Blanchard M, Declercq C, Fabre P, et al Short-term associations between fine and coarse particles and hospital admissions for cardiorespiratory diseases in six French cities. Occup Environ Med. 2008; 65(8): 544–51. 18056749 10.1136/oem.2007.036194

[31] Halonen JI, Lanki T, Yli-Tuomi T, Tiittanen P, Kulmala M, Pekkanen J. Particulate air pollution and acute cardiorespiratory hospital admissions and mortality among the elderly. Epidemiology. 2009; 20(1): 143–53. 10.1097/EDE.0b013e31818c7237 19234403

[32] Halonen JI, Lanki T, Yli-Tuomi T, Kulmala M, Tiittanen P, Pekkanen J. Urban air pollution and asthma and COPD hospital emergency room visits. Thorax. 2008; 63(7): 635–41. 10.1136/thx.2007.091371 18267984

[33] Winquist A, Klein M, Tolbert P, Sarnat SE. Power estimation using simulations for air pollution time-series studies. Environ Health. 2012; 11: 68 10.1186/1476-069X-11-68 22995599 PMC3511883

[34] Samoli E, Stafoggia M, Rodopoulou S, Ostro B, Alessandrini E, Basagaña X, et al Which specific causes of death are associated with short term exposure to fine and coarse particles in Southern Europe? Results from the MED-PARTICLES project. Environ Int. 2014; 67: 54–61. 10.1016/j.envint.2014.02.013 24657768

[35] Ito K, Thurston GD, Silverman RA. Characterization of PM2.5, gaseous pollutants, and meteorological interactions in the context of time-series health effects models. J Expo Sci Environ Epidemiol. 2007; 17 Suppl 2: S45–60. 10.1038/sj.jes.7500627 18079764

[36] Schwartz J, Laden F, Zanobetti. The concentration-response relation between PM(2.5) and daily deaths. Environ Health Perspect. 2002; 110(10): 1025–9. 12361928 10.1289/ehp.021101025 PMC1241029

[37] Beelen R, Raaschou-Nielsen O, Stafoggia M, Andersen ZJ, Weinmayr G, Hoffmann B, et al Effects of long-term exposure to air pollution on natural-cause mortality: an analysis of 22 European cohorts within the multicentre ESCAPE project. Lancet. 2014; 383: 785–95. 10.1016/S0140-6736(13)62158-3 24332274

[38] Samoli E, Analitis A, Touloumi G, Schwartz J, Anderson HR, Sunyer J, et al Estimating the Exposure—Response Relationships between Particulate Matter and Mortality within the APHEA Multicity Project. Environ Health Perspect. 2005; 113(1): 88–95. 15626653 10.1289/ehp.7387 PMC1253715

[39] Wong CM, Ma S, Hedley AJ, Lam TH. Effect of air pollution on daily mortality in Hong Kong. Environ Health Perspect. 2001; 109(4): 335–40. 11335180 10.1289/ehp.01109335 PMC1240272

[40] Cakmak S, Dales RE, Judek S. Do gender, education, and income modify the effect of air pollution gases on cardiac disease? J Occup Environ Med. 2006; 48(1): 89–94. 16404215 10.1097/01.jom.0000184878.11956.4b

[41] Bell ML, Son JY, Peng RD, Wang Y, Dominici F. Brief report: ambient PM_2.5_ and risk of hospital admissions: do risks differ for men and women? Epidemiology. 2015; 26(4): 575–9. 25906368 10.1097/EDE.0000000000000310 PMC4452416

[42] Lv J, Chen W, Sun D, Li S, Millwood IY, Smith M, et al Gender-Specific Association between Tobacco Smoking and Central Obesity among 0.5 Million Chinese People: The China Kadoorie Biobank Study. PLoS One. 2015; 10(4): e0124586 10.1371/journal.pone.0124586 25897789 PMC4405570

[43] Sunyer J, Schwartz J, Tobías A, Macfarlane D, Garcia J, Antó JM. Patients with chronic obstructive pulmonary disease are at increased risk of death associated with urban particle air pollution: a case-crossover analysis. Am J Epidemiol. 2000; 151(1): 50–6. 10625173 10.1093/oxfordjournals.aje.a010121

[44] Qiu H, Tian LW, Pun VC, Ho KF, Wong TW, Yu IT. Coarse particulate matter associated with increased risk of emergency hospital admissions for pneumonia in Hong Kong. Thorax. 2014; 69(11): 1027–33. 10.1136/thoraxjnl-2014-205429 25164925

[45] Goldman GT, Mulholland JA, Russell AG, Strickland MJ, Klein M, Waller LA, et al Impact of exposure measurement error in air pollution epidemiology: effect of error type in time-series studies. Environ Health. 2011; 10: 61 10.1186/1476-069X-10-61 21696612 PMC3146396

[46] Chen Y, Yang Q, Krewski D, Burnett RT, Shi Y, McGrail KM. The effect of coarse ambient particulate matter on first, second, and overall hospital admissions for respiratory disease among the elderly. Inhal Toxicol. 2005; 17(12): 649–55. 16087571 10.1080/08958370500189420

