# Supplementary figures and images for: Fine Particulate Air Pollution and Hospital Emergency Room Visits for Respiratory Disease in Urban Areas in Beijing, China, in 2013
Source: PLoS One. 2016 Apr 7;11(4):e0153099. doi: 10.1371/journal.pone.0153099 (PMC4824441; doi:10.1371/journal.pone.0153099)

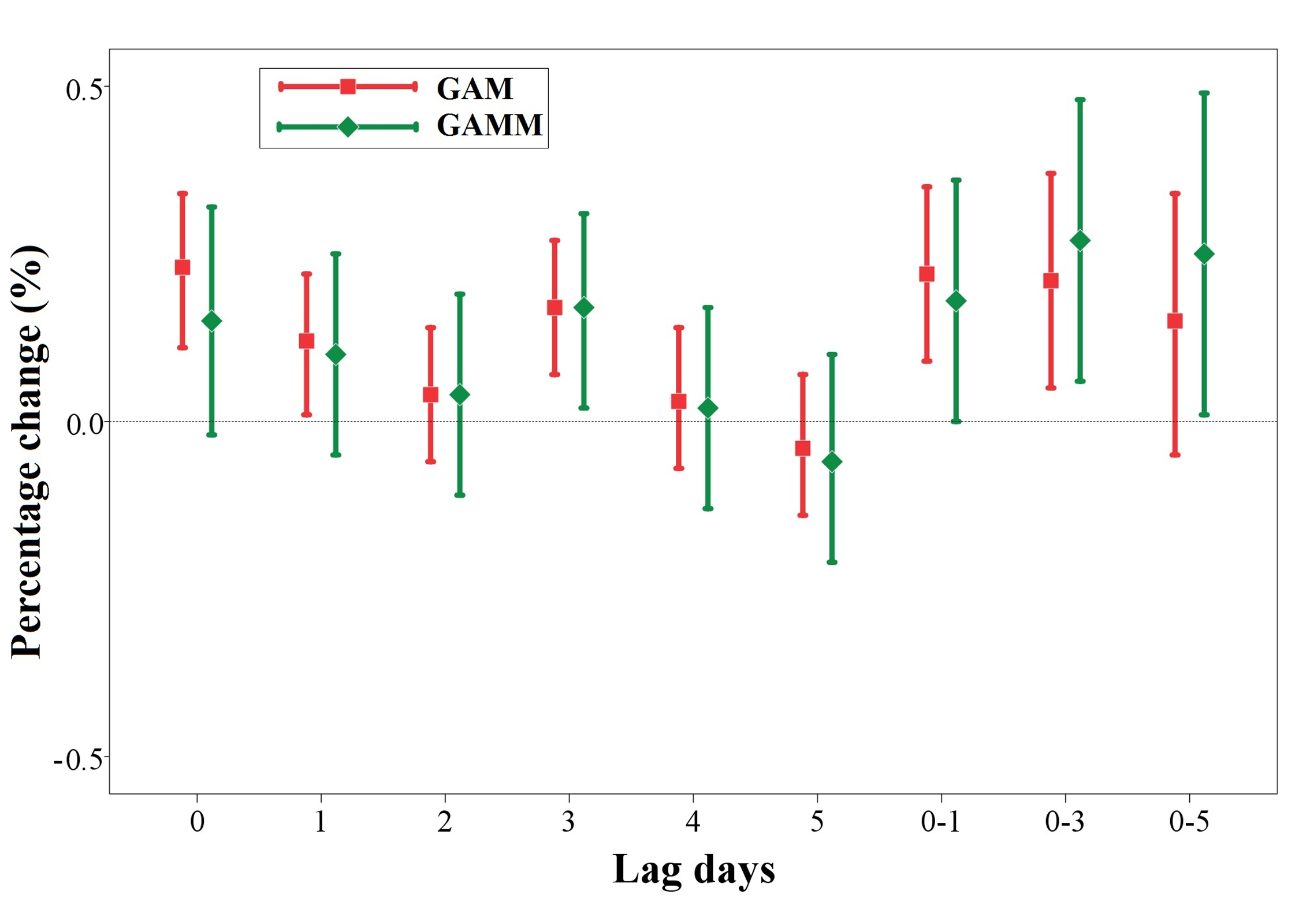

Supplement: S1 Fig — (TIF) [file pone.0153099.s002.tif]
