# Supplementary material for: Fine Particulate Air Pollution and Hospital Emergency Room Visits for Respiratory Disease in Urban Areas in Beijing, China, in 2013
Source: PLoS One. 2016 Apr 7;11(4):e0153099. doi: 10.1371/journal.pone.0153099 (PMC4824441; doi:10.1371/journal.pone.0153099)
Supplement: S1 Table — (DOC) [file pone.0153099.s003.doc]

**S1 Table. Percentage changes with 95% CI in cause-specific respiratory ERV associated with a 10 μg/m^3^ increase in PM_2.5_ concentrations for the different lag structures in two-pollutant models.** *

| Lag  Days | URTI | |  | LRTI | |  | AECOPD | |  | Asthma | |  | |
| --- | --- | --- | --- | --- | --- | --- | --- | --- | --- | --- | --- | --- | --- |
|  | PC | 95%CI |  | PC | 95%CI |  | PC | 95%CI |  | PC | 95%CI |  | |
| Adjusted SO_2_ | | | | | | | | | | | |  | |
| lag_0_ | 0.10 | (-0.11, 0.32) |  | **0.30** | **(0.04, 0.56)** |  | 0.88 | (-0.92, 2.67) |  | -0.10 | (-1.20, 1.00) |  | |
| lag_1_ | 0.16 | (-0.04, 0.36) |  | -0.06 | (-0.30, 0.18) |  | **2.16** | **(0.58, 3.74)** |  | 0.21 | (-0.78, 1.21) |  | |
| lag_2_ | -0.25 | (-0.44, -0.05) |  | 0.20 | (-0.03, 0.44) |  | 1.23 | (-0.35, 2.81) |  | 0.18 | (-0.78, 1.15) |  | |
| lag_3_ | -0.01 | (-0.21, 0.18) |  | 0.16 | (-0.07, 0.40) |  | -0.54 | (-2.19, 1.11) |  | -0.03 | (-0.99, 0.92) |  | |
| lag_4_ | 0.01 | (-0.19, 0.21) |  | 0.09 | (-0.15, 0.33) |  | -1.03 | (-2.76, 0.70) |  | -0.82 | (-1.80, 0.16) |  | |
| lag_5_ | 0.00 | (-0.20, 0.20) |  | -0.10 | (-0.34, 0.14) |  | -0.54 | (-2.30, 1.21) |  | -0.30 | (-1.29, 0.69) |  | |
| lag_0-1_ | 0.13 | (-0.12, 0.37) |  | 0.14 | (-0.16, 0.44) |  | 1.84 | (-0.17, 3.84) |  | 0.10 | (-1.12, 1.33) |  | |
| lag_0-3_ | -0.03 | (-0.35, 0.30) |  | 0.16 | (-0.23, 0.55) |  | 1.26 | (-1.40, 3.91) |  | 0.08 | (-1.51, 1.66) |  | |
| lag_0-5_ | -0.04 | (-0.43, 0.36) |  | 0.13 | (-0.33, 0.60) |  | 0.43 | (-2.82, 3.68) |  | -1.06 | (-2.97, 0.85) |  | |
| Adjusted O_3_ | | | | | | | | | | | |  | 0.21 |
| lag_0_ | **0.19** | **(0.02, 0.35)** |  | **0.32** | **(0.11, 0.53)** |  | **1.94** | **(0.53, 3.35)** |  | -0.64 | (-1.54, 0.27) |  | |
| lag_1_ | 0.13 | (-0.02, 0.28) |  | 0.05 | (-0.14, 0.23) |  | **2.41** | **(1.21, 3.61)** |  | 0.18 | (-0.61, 0.96) |  | |
| lag_2_ | -0.13 | (-0.27, 0.02) |  | **0.18** | **(0.01, 0.36)** |  | 1.07 | (-0.12, 2.27) |  | 0.05 | (-0.70, 0.80) |  | |
| lag_3_ | 0.13 | (-0.02, 0.27) |  | 0.16 | (-0.02, 0.34) |  | 0.84 | (-0.40, 2.08) |  | -0.01 | (-0.75, 0.74) |  | |
| lag_4_ | 0.06 | (-0.09, 0.21) |  | 0.10 | (-0.08, 0.28) |  | -1.09 | (-2.41, 0.24) |  | -0.56 | (-1.32, 0.20) |  | |
| lag_5_ | -0.05 | (-0.20, 0.10) |  | -0.03 | (-0.22, 0.15) |  | -1.51 | (-2.87, -0.16) |  | -0.59 | (-1.37, 0.18) |  | |
| lag_0-1_ | **0.20** | **(0.02, 0.37)** |  | **0.25** | **(0.02, 0.47)** |  | **2.91** | **(1.40, 4.42)** |  | -0.24 | (-1.21, 0.74) |  | |
| lag_0-3_ | 0.19 | (-0.03, 0.41) |  | 0.18 | (-0.09, 0.46) |  | **2.95** | **(1.12, 4.78)** |  | 0.07 | (-1.08, 1.22) |  | |
| lag_0-5_ | 0.10 | (-0.18, 0.38) |  | 0.21 | (-0.13, 0.55) |  | 1.31 | (-1.06, 3.68) |  | -0.65 | (-2.08, 0.78) |  | |
| Adjusted CO | | | | | | | | | | | |  | |
| lag_0_ | **0.25** | **(0.04, 0.46)** |  | 1.31 | (-0.38, 3.00) |  | 0.14 | (-0.97, 1.25) |  | 0.42 | (-1.62, 2.46) |  | |
| lag_1_ | **0.19** | **(0.00, 0.39)** |  | **2.22** | **(0.70, 3.75)** |  | 0.08 | (-0.88, 1.03) |  | 0.14 | (-1.88, 2.17) |  | |
| lag_2_ | -0.02 | (-0.21, 0.17) |  | 0.82 | (-0.71, 2.34) |  | 0.71 | (-0.30, 1.72) |  | 0.76 | (-1.23, 2.74) |  | |
| lag_3_ | 0.13 | (-0.06, 0.32) |  | 1.56 | (-0.27, 3.39) |  | -0.06 | (-0.98, 0.86) |  | 1.51 | (-0.47, 3.49) |  | |
| lag_4_ | 0.15 | (-0.05, 0.35) |  | -0.12 | (-2.11, 1.86) |  | 0.35 | (-0.68, 1.38) |  | 0.73 | (-1.30, 2.75) |  | |
| lag_5_ | -0.13 | (-0.33, 0.06) |  | -0.29 | (-2.32, 1.74) |  | 0.09 | (-0.92, 1.11) |  | 1.93 | (-0.08, 3.93) |  | |
| lag_0-1_ | **0.33** | **(0.06, 0.59)** |  | **2.15** | **(0.04, 4.26)** |  | 0.38 | (-0.94, 1.69) |  | 0.22 | (-1.95, 2.39) |  | |
| lag_0-3_ | **0.51** | **(0.14, 0.88)** |  | 2.58 | (-0.47, 5.62) |  | 0.91 | (-0.92, 2.74) |  | 0.79 | (-1.63, 3.21) |  | |
| lag_0-5_ | 0.44 | (-0.03, 0.90) |  | 2.56 | (-1.32, 6.45) |  | 1.38 | (-0.86, 3.62) |  | 1.50 | (-1.33, 4.33) |  | |
| Adjusted NO_2_ | | | | | | | | | | | |  | |
| lag_0_ | 0.06 | (-0.16, 0.29) |  | 0.15 | (-0.13, 0.42) |  | 1.32 | (-0.61, 3.26) |  | -0.63 | (-1.81, 0.56) |  | |
| lag_1_ | **0.23** | **(0.01, 0.44)** |  | 0.03 | (-0.23, 0.29) |  | 0.91 | (-0.87, 2.68) |  | 0.69 | (-0.39, 1.77) |  | |
| lag_2_ | -0.05 | (-0.27, 0.16) |  | **0.31** | **0.05, 0.56)** |  | 0.68 | (-1.11, 2.47) |  | 0.20 | (-0.86, 1.26) |  | |
| lag_3_ | 0.17 | (-0.04, 0.38) |  | **0.29** | **(0.03, 0.54)** |  | -0.54 | (-2.38, 1.30) |  | 0.75 | (-0.29, 1.80) |  | |
| lag_4_ | 0.08 | (-0.14, 0.29) |  | 0.04 | (-0.22, 0.30) |  | -2.13 | (-4.06, -0.20) |  | -0.63 | (-1.70, 0.43) |  | |
| lag_5_ | 0.20 | (-0.01, 0.42) |  | 0.09 | (-0.17, 0.36) |  | -0.97 | (-2.92, 0.98) |  | -0.31 | (-1.39, 0.77) |  | |
| lag_0-1_ | 0.18 | (-0.08, 0.44) |  | 0.09 | (-0.23, 0.41) |  | 1.23 | (-0.98, 3.43) |  | 0.17 | (-1.16, 1.49) |  | |
| lag_0-3_ | 0.31 | (-0.03, 0.65) |  | 0.22 | (-0.20, 0.63) |  | 0.79 | (-2.06, 3.65) |  | 0.90 | (-0.79, 2.59) |  | |
| lag_0-5_ | 0.40 | (-0.02, 0.82) |  | 0.28 | (-0.22, 0.79) |  | -0.77 | (-4.32, 2.77) |  | 0.18 | (-1.85, 2.20) |  | |

*statistically significant results at the 5% level are indicated in bold; PC-percentage change.
