# Supplementary material for: Fine Particulate Air Pollution and Hospital Emergency Room Visits for Respiratory Disease in Urban Areas in Beijing, China, in 2013
Source: PLoS One. 2016 Apr 7;11(4):e0153099. doi: 10.1371/journal.pone.0153099 (PMC4824441; doi:10.1371/journal.pone.0153099)
Supplement: S2 Table — (DOC) [file pone.0153099.s004.doc]

**S2 Table. Percentage changes with 95% CI in total respiratory ERV associated with a 10 μg/m^3^ increase in the PM_2.5_ concentrations at lag_0_ by different degrees of freedom (*df*) for time, temperature, and relative humidity.**

| **Degrees of freedom** | | | PC | 95%CI | *P* value | R square | UBRE^#^ |
| --- | --- | --- | --- | --- | --- | --- | --- |
| Time | Temperature | Relative Humidity |
| **11** | **3** | **3** | **0.23** | **(0.11, 0.34)** | **<0.001** | **0.884** | **0.936** |
| 6 | 3 | 3 | 0.41 | (0.29, 0.52) | <0.001 | 0.736 | 3.182 |
| 7 | 3 | 3 | 0.28 | (0.17, 0.39) | <0.001 | 0.842 | 1.493 |
| 8 | 3 | 3 | 0.22 | (0.11, 0.34) | <0.001 | 0.824 | 1.839 |
| 9 | 3 | 3 | 0.22 | (0.10, 0.33) | <0.001 | 0.875 | 1.060 |
| 10 | 3 | 3 | 0.22 | (0.11, 0.34) | <0.001 | 0.884 | 0.940 |
| 12 | 3 | 3 | 0.22 | (0.10, 0.33) | <0.001 | 0.885 | 0.928 |
| 11 | 2 | 3 | 0.22 | (0.11, 0.34) | <0.001 | 0.885 | 0.933 |
| 11 | 4 | 3 | 0.23 | (0.11, 0.35) | <0.001 | 0.884 | 0.938 |
| 11 | 5 | 3 | 0.23 | (0.12, 0.35) | <0.001 | 0.884 | 0.945 |
| 11 | 6 | 3 | 0.23 | (0.11, 0.35) | <0.001 | 0.884 | 0.939 |
| 11 | 3 | 2 | 0.23 | (0.12, 0.35) | <0.001 | 0.884 | 0.946 |
| 11 | 3 | 4 | 0.23 | (0.11,0.34) | <0.001 | 0.884 | 0.941 |
| 11 | 3 | 5 | 0.22 | (0.11, 0.34) | <0.001 | 0.884 | 0.944 |
| 11 | 3 | 6 | 0.22 | (0.10, 0.33) | <0.001 | 0.885 | 0.929 |

^#^UBRE, un-biased risk estimator.

Note: The *df* value used in our study model are indicated in bold; PC-percentage change.
