# Supplementary material for: Fine Particulate Air Pollution and Hospital Emergency Room Visits for Respiratory Disease in Urban Areas in Beijing, China, in 2013
Source: PLoS One. 2016 Apr 7;11(4):e0153099. doi: 10.1371/journal.pone.0153099 (PMC4824441; doi:10.1371/journal.pone.0153099)
Supplement: S3 Table — (DOC) [file pone.0153099.s005.doc]

**S3 Table. Percentage changes with 95% CI in total respiratory ERV associated with a 10 μg/m^3^ increase in PM_2.5_ concentrations when the current day’s temperature and 14-day moving average temperature were controlled.**

| Lag days | Temperature0 | |  | Temperature14 | |
| --- | --- | --- | --- | --- | --- |
|  | Percentage change(95%CI) | *P* value |  | Percentage change(95%CI) | *P* value |
| lag_0_ | 0.23 (0.11, 0.34) | <0.001 |  | 0.24 (0.13, 0.35) | <0.001 |
| lag_1_ | 0.12 (0.01, 0.22) | 0.029 |  | 0.15 ( 0.05, 0.26) | 0.003 |
| lag_2_ | 0.04 (-0.06.0.14) | 0.436 |  | 0.04 (-0.06, 0.14) | 0.388 |
| lag_3_ | 0.17 (0.07, 0.27) | 0.001 |  | 0.16 (0.06, 0.27) | 0.001 |
| lag_4_ | 0.03 (-0.07,0.14) | 0.518 |  | 0.04 (-0.07, 0.14) | 0.471 |
| lag_5_ | -0.04 (-0.14, 0.07) | 0.510 |  | -0.02 (-0.13, 0.08) | 0.687 |
| lag_0-1_ | 0.22 (0.09, 0.35) | 0.001 |  | 0.25 (0.13, 0.38) | <0.001 |
| lag_0-3_ | 0.21 (0.05, 0.37) | 0.009 |  | 0.28 (0.13, 0.43) | <0.001 |
| lag_0-5_ | 0.15 (-0.05, 0.34) | 0.151 |  | 0.24 (0.05, 0.43) | 0.014 |

Note: Temperature0, the current day’s temperature was controlled; and Temperature14, 14-day moving average temperature was controlled.
