# Supplementary material for: Fine Particulate Air Pollution and Hospital Emergency Room Visits for Respiratory Disease in Urban Areas in Beijing, China, in 2013
Source: PLoS One. 2016 Apr 7;11(4):e0153099. doi: 10.1371/journal.pone.0153099 (PMC4824441; doi:10.1371/journal.pone.0153099)
Supplement: S4 Table — (DOC) [file pone.0153099.s006.doc]

**S4 Table. Percentage changes with 95% CI in cause-specific respiratory ERV associated with a 10 μg/m^3^ increase in PM_2.5_ concentrations for different lag structures in single-pollutant GAMM.** *

| Lag  Days | URTI | |  | LRTI | |  | AECOPD | |  | Asthma | |
| --- | --- | --- | --- | --- | --- | --- | --- | --- | --- | --- | --- |
|  | PC | 95%CI |  | PC | 95%CI |  | PC | 95%CI |  | PC | 95%CI |
| lag_0_ | 0.11 | (-0.10, 0.32) |  | **0.29** | **(0.03, 0.55)** |  | 0.64 | (-0.75, 2.02) |  | -0.49 | (-1.39, 0.41) |
| lag_1_ | 0.12 | (-0.08, 0.31) |  | 0.09 | (-0.15, 0.32) |  | **1.64** | **(0.43, 2.85)** |  | 0.06 | (-0.72, 0.85) |
| lag_2_ | -0.09 | (-0.28, 0.10) |  | **0.25** | **(0.02, 0.47)** |  | 0.85 | (-0.33, 2.04) |  | 0.04 | (-0.72, 0.80) |
| lag_3_ | 0.14 | (-0.04, 0.33) |  | **0.23** | **(0.00, 0.45)** |  | 0.69 | (-0.54, 1.91) |  | -0.06 | (-0.80,0.69) |
| lag_4_ | 0.02 | (-0.17, 0.21) |  | 0.04 | (-0.19, 0.27) |  | -0.70 | (-2.00, 0.59) |  | -0.17 | (-0.92, 0.58) |
| lag_5_ | -0.03 | (-0.22, 0.17) |  | -0.09 | (-0.33, 0.15) |  | -1.45 | (-2.79, -0.11) |  | -0.12 | (-0.89, 0.64) |
| lag_0-1_ | 0.14 | (-0.08, 0.36) |  | **0.29** | **(0.02, 0.57)** |  | 1.40 | (-0.05, 2.84) |  | -0.28 | (-1.22, 0.66) |
| lag_0-3_ | 0.16 | (-0.10, 0.42) |  | **0.52** | **(0.20, 0.83)** |  | **2.15** | **(0.46, 3.85)** |  | -0.37 | (-1.44, 0.70) |
| lag_0-5_ | 0.13 | (-0.17, 0.43) |  | **0.50** | **(0.14, 0.87)** |  | 1.56 | (-0.41, 3.52) |  | -0.70 | (-1.92, 0.52) |

*statistically significant results at the 5% level are indicated in bold; PC-percentage change. GAM, the estimated effects in a GAM using mean PM_2.5_ concentrations from 17 monitoring sties; GAMM, the estimated effects in GAMM using assigned PM_2.5_ concentrations for each hospital.
